# Full-Wave Simulation of a Solenoid RF Coil for Small Animal Magnetic Resonance Imaging with a Clinical Scanner

**DOI:** 10.3390/s25092673

**Published:** 2025-04-23

**Authors:** Giulio Giovannetti, Francesca Frijia, Alessandra Flori, Vincenzo Positano

**Affiliations:** 1 CNR Institute of Clinical Physiology, 56124 Pisa, Italy; giulio.giovannetti@cnr.it; 2 Bioengineering Unit, Fondazione Toscana G. Monasterio, 56124 Pisa, Italy; ffrijia@ftgm.it (F.F.); alessandra.flori@ftgm.it (A.F.)

**Keywords:** radiofrequency coils, magnetic resonance, inductance, sample resistance, numerical simulations

## Abstract

Clinical research groups rarely have easy access to dedicated animal Magnetic Resonance (MR) systems. For this reason, dedicated hardware has to be developed to optimize small animal imaging on clinical scanners. In MR systems, radiofrequency (RF) coils are key components in the acquisition process of the MR signal, and the design of hand-crafted, organ-specific RF coils can be a constraint in many research projects. Accurate design and simulation processes enable the optimization of RF coil performance for a given application by avoiding trial-and-error approaches. This paper describes the full-wave simulation of a solenoidal coil for Magnetic Resonance Imaging (MRI) using the finite-difference time-domain (FDTD) method. Such a simulator enables the estimation of the coil’s magnetic field pattern in a loaded condition, the coil inductance, and the sample-induced resistance. The resulting accuracy is verified with data acquired with a solenoid prototype designed for small animal experiments with a 3T MRI clinical scanner.

## 1. Introduction

Magnetic Resonance Imaging (MRI) is a 3D, non-invasive imaging technique that produces detailed anatomical images. MRI uses a spectroscopic technique to obtain accurate chemical and physical information about molecules. Most commonly, protons (^1^H) are imaged, although carbon (^13^C), phosphorus (^31^P), sodium (^23^Na), and fluorine (^19^F) are also of significant interest. The time required for a typical imaging study ranges from 1 to 10 min, whereas new fast imaging techniques can acquire images in less than 50 milliseconds. MRI research involves fundamental tradeoffs between resolution, imaging time, and signal-to-noise ratio (SNR), which depend heavily on hardware performance and innovations.

The subject undergoing an MRI exam is placed in a static magnetic field B0. At equilibrium conditions, nuclear spins with nonzero magnetic moment are aligned along B0, processing around the direction of B0 at the Larmor frequency, defined as the product of the nucleus gyromagnetic ratio (γ) and the static field strength (B0). Nuclear spin alignment provides the net magnetization that generates the MRI signal. Low magnetic field strengths typically employed in clinical practice (i.e., 0.2–3T) are associated with a Larmor frequency in the range of units to tens of MHz, falling within the radiofrequency (RF) range. An RF transmit coil generates an excitation in the transverse plane (perpendicular to the main B0 field direction) that induces an oscillation at the Larmor frequency (resonance condition) of the chemical species of interest. The generated B1 magnetic field induces a signal in a receiving RF coil (i.e., free induction decay (FID)). The FID time signal undergoes a Fourier transformation to obtain a spectrum in the frequency domain. To localize the source of the MRI signal, magnetic field gradients are applied to obtain spatially selective excitation and/or spatial encoding. Magnetic field gradients are spatially varying magnetic fields inducing small modifications (i.e., RF bandwidth) of the Larmor frequency depending on the spatial coordinate. The Larmor frequency of spins depends linearly on the spatial location along the direction of the gradient. With the application of gradient excitations, only the spins in a slice whose resonance frequency falls into the RF bandwidth are excited. The RF excitation rotates a portion of the magnetization (i.e., flip angle) to the transverse plane, which a receiving RF coil can detect.

The transmit coil should generate a highly uniform RF magnetic field in a wide field of view (FOV), as the extension of the region being analyzed is not known a priori. Therefore, transmit coils are usually large to optimize the magnetic field homogeneity and include a significant tissue volume. On the other hand, the receiving RF coil should maximize the SNR. Hence, the receive coil dimensions should be minimized [1]. Due to the tradeoff between transmit and receive coils needs, the transmit and receive coils design should be adapted to the specific goal and the sample dimensions. Clinical scanners have a typical bore diameter of around 0.5/1 m and RF coil size adapted to the human body. This setting is not optimal for performing MR experiments in small animal models, such as mice and rats. Hence, the design and development of dedicated RF coils are crucial for optimizing the signal-to-noise ratio (SNR) and spatial resolution.

A significant example of a dedicated RF coil design is reported in [2]. A dual-tuned RF coil configuration for MR imaging experiments on mice using a 3T clinical scanner was presented, consisting of a 1H whole-body volume RF coil with a solenoidal geometry for anatomical imaging of the mouse. In the study, a 13C single circular loop surface RF coil was also tested, built for MR metabolic studies with hyperpolarized tracers (in particular, hyperpolarized [1-13C]pyruvate) at 3T, an advanced MR application demanding high sensitivity for detection of the spectroscopic signal from the different 13C-labeled metabolites [3,4,5,6]. Several aspects, such as simulation, fabrication, workbench characterization, and in vivo testing of the coils, were addressed in the article. Proper anatomical details could be achieved with the 1H solenoid. The results reported in the paper confirm that the proposed RF coil configuration was fully compatible and could be easily integrated with the MR clinical scanner to achieve good image quality in small animal studies using a clinical setting [7]. A solution such as the one proposed can thus emerge as a simple and cost-effective alternative for clinical research groups working with small animal experimental models that lack access to a dedicated preclinical scanner. In particular, the interconnection to the scanner is achieved via 50 Ω coaxial cables, and coils must be matched to 50 Ω to optimize the energy transfer through all parts of the spectrometer [8]. Finally, for coils designed for the transmit/receive mode with clinical scanners, a T/R switch must be inserted between the coil and the scanner, which also monitors the specific absorption rate (SAR) to prevent dangerous tissue heating [9].

The solenoid employed in a previous paper [2] was designed and simulated with the magnetostatic numerical integration of the Biot–Savart law, which calculates the B1 field distribution in free space (unloaded coil). This simulation approach, which is accurate for low-frequency MR applications, cannot accurately estimate the actual magnetic field distribution inside the sample and does not permit a proper SNR calculation necessary to consider sample noise. Due to this limitation, a long trial-and-error procedure was needed to obtain an optimal coil design. For a more effective MR experiment simulation, full-wave simulations based on the finite-difference time-domain (FDTD) algorithm [10] can be employed, enabling the modeling and prediction of coil performance before physical prototyping, thereby saving time and resources. 

This method has been widely used in various MRI applications. For example, Wang et al. [11] employed the FDTD algorithm for SAR and temperature calculations for the human head in a volume coil, Chen et al. [12] estimated B1 field and SAR inside the human head generated by a shielded birdcage coil, and Giovannetti et al. [13] optimized the overlap distance between the two elliptical loops of a phased-array coil.

In this paper, we describe the retrospective simulation of a solenoid using the FDTD method, which enables the estimation of the B1 magnetic field pattern for the coil in loaded conditions by considering how the magnetic field is affected by the sample’s electromagnetic properties. Moreover, since sample losses are dominant with respect to coil losses at high magnetic field strengths [1], the sample-induced resistance was estimated by exploiting the fact that the FDTD method also provides simulation data in the time domain. Since the knowledge of the sample-induced resistance, the coil inductance, and the magnetic field pattern in loaded conditions permits a complete characterization of coil performance, the objective of this work is to demonstrate the ability of electromagnetic simulation to predict coil image homogeneity and SNR.

## 2. Materials and Methods

**Figure 1:**
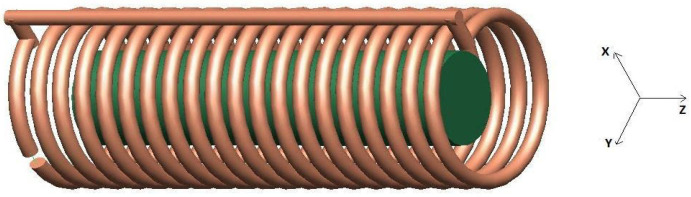
CAD model of the loaded solenoid for FDTD simulation. A circular cross-section (diameter 4 mm) wire conductor was used with 20 turns (spacing between turns 6 mm). The solenoid has a length of 12 cm and a diameter of 4.4 cm. A 10 cm length, 2.5 cm diameter cylindrical phantom was placed at the center of the solenoid on the solenoid axis.

A solenoid coil designed to accommodate small animals such as mice for MRI experiments was simulated. A unique copper wire cylindrical winding (20 turns, spacing between turns *p* = 6 mm) constituted the coil (Figure 1). The solenoid had a length of H = 12 cm and a diameter of D = 4.4 cm to achieve a uniform MR field within the FOV of interest (more details can be found in [2]). The load was a homogeneous cylindrical phantom (10 cm length, 2.5 cm diameter) placed at the center of the solenoid on the solenoid axis. The phantom dielectric properties (σ = 0.6 S/m, ɛ = 80) meet the American Society for Testing and Materials (ASTM) criteria for MR phantom development [14]. The development steps included FDTD simulation for inductance, sample-induced resistance calculation, and magnetic field evaluation. The developed coil was tested in a 3T MRI clinical scanner.

### 2.1. FDTD Simulations

The full-wave simulations were performed with the FDTD algorithm using the commercially available software XFdtd 7.8 (Remcom, State College, PA, USA). The solenoid was designed utilizing the geometry workspace of the XFdtd tool; in the simulations, we assumed the solenoid to be constituted by a Perfect Electric Conductor (PEC) (Figure 1). An adaptive non-uniform mesh, finer in the proximity of the solenoid conductor, was used to minimize computational time and memory load while achieving good accuracy. Finally, Perfect Matched Layer (PML) boundary conditions were used [15] for truncating outward waves and mimicking an infinite computational domain.

Curved conductor surfaces were accurately modeled using a tool that employs geometric information to provide a computational domain subcellular discretization, thereby increasing simulation accuracy for a given grid resolution. This approach reduces memory constraints and overall simulation time while maintaining a desired level of accuracy. The simulations were performed using automatic convergence detection, set to −90 dB, ensuring complete energy decay so that the signal decayed sufficiently.

### 2.2. Inductance and Magnetic Field Evaluation

The inductance of the simulated solenoid was measured using a tuning capacitor C inserted into a 4 mm opening, resulting in a resonant circuit fed by an ideal current port. A damped voltage oscillation was induced on the capacitor using a Gaussian derivative pulse as the input excitation. It allowed the evaluation of resonance frequency and solenoid inductance. The value of the tuning capacitor was much larger than the parasitic capacitances, estimated with a semi-empirical formula [16,17].

Finally, the magnetic field pattern of the loaded solenoid was evaluated using a current feed with a sinusoidal waveform (amplitude 1 A, frequency 128 MHz).

### 2.3. Sample-Induced Resistance Evaluation

The sample-induced resistance of the coil was evaluated based on the resonant circuit theory [18]. The loaded coil quality factor *Q* was calculated as the ratio between the energy stored and lost at the *i*th cycle. In detail, the coil was perturbed by a Gaussian pulse, inducing a voltage oscillation damped by the losses. Then, the *Q* value was calculated, knowing that the energy stored by a capacitor is proportional to the square of the voltage across it [18]:

(1) $$Q=2\pi \frac{V_{i}^{2}}{V_{i}^{2}-V_{i+1}^{2}},$$

where $V_{i}$ and $V_{i+1}$ are the voltage at *i*th and at (*i* + 1)th cycles. The solenoid simulation was performed with a PEC conductor, so the coil resistance completely vanishes, and the sample dissipated all the energy. Hence, the sample-induced resistance $R_{sample}$ can be evaluated as

(2) $$R_{sample}=\frac{2\pi f_{0}L}{Q},$$

where $f_{0}$ is the resonant frequency and L is the solenoid inductance.

### 2.4. MR Acquisitions with the Solenoid Prototype

**Figure 2:**
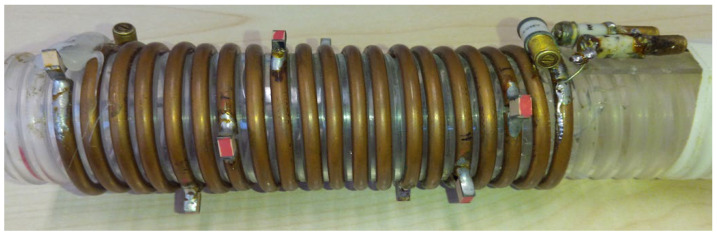
Picture of the homemade solenoid prototype. A circular cross-section wire conductor (diameter 4 mm) was wrapped around a plexiglass cylindrical support with 20 turns, spaced 6 mm apart. The solenoid has a length of 12 cm and a diameter of 4.4 cm.

The solenoid, built on a plexiglass cylindrical support (Figure 2), was tuned at 127.75 MHz and matched to 50 Ω using fixed (ATC 100C—American Technical Ceramics, Huntington Station, NY, USA) and variable (AP40HV Voltronics, Chicago, IL, USA) capacitors, as detailed in [2].

A cylindrical phantom (10 cm length, 2.5 cm diameter) containing 10 g [1-^13^C] sodium (Na) acetate, 58 mL H_2_O, and 0.5 mmol Dotarem was placed into the solenoid. The phantom composition was optimized to maximize the MR signal quality.

Phantom images were acquired with a 3T GE HDx TWINSPEE (GE Healthcare, Waukesha, WI) clinical scanner, using a Gradient Echo (GRE) sequence (FOV = 120 mm, 160 × 160 matrix, slice thickness = 5 mm, TE/TR = 3.40/800 ms). The double-angle method (DAM) [19], which requires the acquisitions of two images with different nominal flip-angles (45° and 90° in our acquisitions), was used to evaluate the B1 map, which was computed using OsiriX 6.5 software (Pixmeo, Geneva, Switzerland) [20].

## 3. Results

The simulation method described in Section 2.2 yielded an inductance value of L = 5.03 µH, which is very similar to the value (5.26 µH) obtained by the integral method [2], the implementation of which for the solenoid is fully detailed in [21]. Sample-induced resistance from numerical simulations using Equation (2) resulted in 24 Ω.

**Figure 3:**
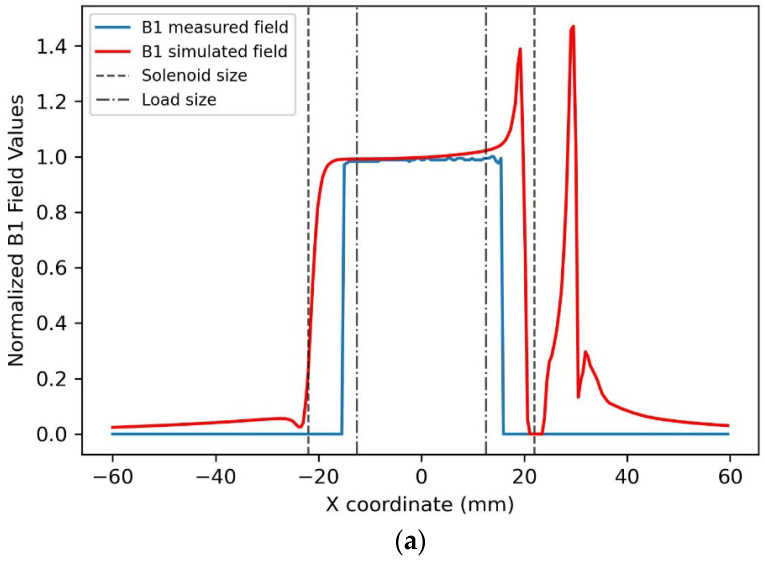

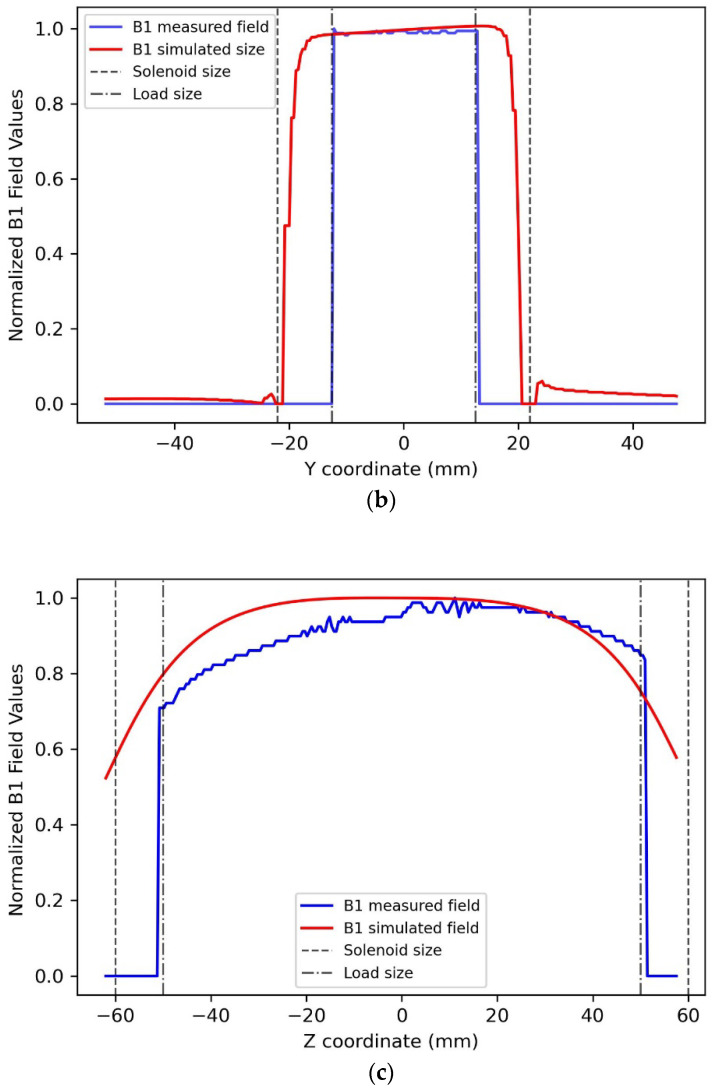
Simulated B1 magnetic field profiles obtained for the 1H solenoid and measured B1 field profiles extracted from phantom images along the x (**a**), y (**b**), and z (**c**) axes. B1 fields were normalized to their values at the solenoid center.

The modulus profiles of the magnetic field along three straight lines passing through the solenoid center were estimated for the x, y, and z axes (Figure 3). In particular, the blue lines are obtained by extracting the profiles along each axis in the cylindrical phantom B1 mapping acquisition. In contrast, the red lines refer to the simulated magnetic field. Both profiles were normalized to the maximum intensity of the B1 map and simulated magnetic field, respectively. The solenoid and phantom sizes are shown in the same figure as black dotted lines.

**Figure 4:**
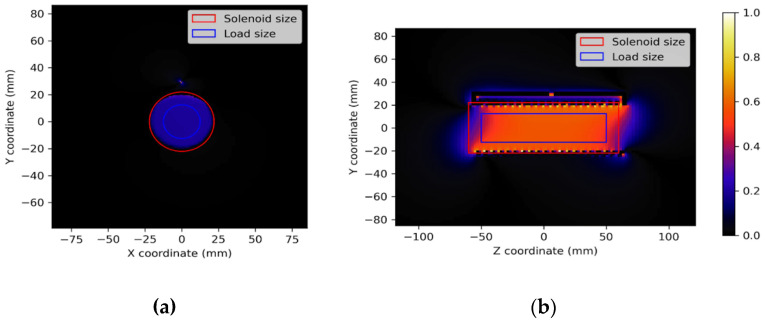
B_1_ maps on transversal (**a**) and longitudinal plane (**b**).

The homogeneity of profile P was evaluated on the load size as Hp = 100 * (max(P) − min(P))/max(P), as reported in Table 1. Figure 4 shows the magnetic field distribution among transversal and longitudinal planes.

## 4. Discussion

In biomedical research, many experimental studies utilize small animal models to gain a deeper understanding of complex human diseases by collecting in vivo anatomical, functional, and metabolic information. Among the available imaging techniques, MRI has several characteristics that make it an ideal candidate for small animal imaging, including 3D capabilities, high contrast, high spatial and temporal resolution, and a high safety profile. Small-bore high-field MR scanners (field strengths between 4.7 and 21 T) are the gold standard equipment for small animal MRI. However, these systems are expensive and not readily available in many research centers. Clinical whole-body MRI systems offer a more cost-effective and widely available alternative to dedicated preclinical systems for conducting studies in small animal models [22,23,24].

Clinical MRI systems operate at lower field strengths (1.5 and 3.0 T), limiting the achievable SNR and spatial resolution. However, several studies demonstrated that high-resolution MR imaging in rodents can be reliably performed using a clinical scanner [25,26,27]. The design of dedicated RF coils represents a fundamental step for effective MR small-animal imaging in clinical scanners. For instance, in Pillai et al., a custom-built four-channel phased array coil was used for the study of central nervous system pathologies in rats using a clinical 3T scanner [28], while in Rad et al. dedicated small animal volume coils were used for in vivo detection of prostate tumor in mice at 3T using a clinical scanner [29].

In particular, volume coils, such as the birdcage or solenoid coils [30], can provide a homogeneous sensitivity profile over the whole animal, making whole-body imaging possible [31]. In Stara et al. [32], a custom-built birdcage coil for mice experiments in a human 7T scanner is described.

This study describes the design, development, and testing of a solenoid RF coil optimized for MRI studies in small animals that can be easily integrated with a clinical 3T scanner.

An accurate simulation process was necessary to avoid trial-and-error approaches during the design of the RF solenoidal coil. To date, the FEM-based and the FDTD-based simulation software are the most employed for birdcage RF coil design [31]. In this study, simulations were performed using the FDTD method, a versatile and highly efficient computational method that allows for a comprehensive characterization of RF coil performance and properties for MRI purposes, as well as the modeling of different RF coil configurations over a wide frequency range (>500 MHz). For instance, in Ibrahim et al., numerical simulations using FDTD were reported to determine the behavior of the transmit and receive B1 fields produced by the RF coil at magnetic field strengths ranging from 1.5 T to 8 T [33], while in [34] different birdcage coil configurations were simulated and compared for small animal MRI at various magnetic field strength ranging from 1.5 to 11.7 T. The same research group [35] employed a commercially available simulation software based on FDTD for the electromagnetic-field analysis (in terms of sensitivity, homogeneity, and safety) of a hybrid-type birdcage coil to determine the optimal coil specification for application at ultra-high field MR (at 11.7 T).

Among other advantages, the FDTD method enables modeling the effect induced by biological tissue (including the various anatomical districts of the human body) on the RF coils’ performance, taking into account field-tissue interactions. In Song et al., the FDTD simulation method was used to improve a volume coil design for optimized brain MRI at 4.7 T with a human model [36]; in Wang et al. [37], FDTD was used to simulate the human body in combination with the finite-element time-domain (FETD) method in the simulation of the RF coil for MRI applications. A few other studies proposed combining the FDTD method with other computational techniques, such as the Method of Moments (MoM), to provide a hybrid simulation platform in the field of RF coil design for MRI applications [38,39,40].

In this paper, the complete RF coil design included the computation of the inductance, the magnetic field pattern in the loaded configuration, and the sample losses. The solenoid inductance simulation result was compared with analytical calculation data obtained with the integral method, resulting in an error of 4.4%. The simulator allowed the estimation of the sample-induced resistance by using a validated algorithm based on resonant circuit theory [41,42].

The solenoid magnetic field pattern, which can be observed in Figure 3, is very homogeneous along the X and Y coil axes. The homogeneity in the FOV of interest is high, corresponding to the B1 field among the simulated/images load that mimics the animal size (Table 1). In the X-axis profile, we can see the presence of the “hot spot” magnetic field generated by the “reclosing conductor”, which does not influence the homogeneity of the magnetic field in the useful FOV. A good homogeneity is also observable in the Z-axis direction (Table 1). By comparing the plots of the simulated magnetic field for the loaded coil with those obtained from the experimental acquisition, it is possible to verify the great accuracy of the full-wave simulation.

The proposed simulator can be used to design coils with different geometries, including the effect of a specific load. The simulation allows the incorporation of a phantom or a part of a human/animal body in the computational space, even by importing CAD files into the tool project. Hence, an accurate sample–coil interaction model can be obtained at all frequencies of interest in MRI/MRS applications. This approach could dramatically speed up the developing process, avoiding the production of several physical prototypes.

This study used wire (cylindrical rod) conductors to simulate and build the solenoid. The proposed approach could be extended to the simulation of coils constituted by rectangular strips used for coil building. Interestingly, it is possible to use the relation between the “equivalent width” of a rectangular strip and the radius of a wire [43] to obtain the inductance value without needing a new simulation.

Some limitations can be recognized in the study. We retrospectively demonstrated how electromagnetic simulations can permit a comprehensive characterization of coil performance for a previously developed coil optimized by standard design methods and tested in a clinical MRI scanner. The obtained findings can serve as a starting point for the prospective design of new coil structures. However, at this stage, we have not performed a perspective design and validation of new coils. Complete coil design using full-wave simulation could require a long computational time to achieve high spatial resolution of the computational domain, limiting the number of coil structures that can be explored. The use of high-performance computers may be necessary for effective coil design.

## 5. Conclusions

In conclusion, this work proposes using a full-wave FDTD-based simulator to design a 1H solenoid for small animal experiments with a clinical MR scanner. The proposed tool can also be useful for designing and simulating different and more complicated geometry coils effectively before manufacturing. The tool could be useful in the entire RF spectrum employed in MR. The accuracy of the performed simulations was verified by comparison with analytical calculation and experimental results. Such tool potential permits to perform simulations of more realistic MR experiments, although at the expense of longer computational time (hours).

## Figures and Tables

**Table 1:** Homogeneity valued for simulated magnetic field and measured B1 field.

Profile	Simulated Magnetic Field Homogeneity (%)	Measured B_1_ Field Homogeneity (%)
X	98.25	99.16
Y	98.50	98.25
Z	76.15	76.18

## Data Availability

Data are contained within the article.
